# Prognostic value of preablative stimulated thyroglobulin and its decline after ^131^I therapy in differentiated thyroid cancer

**DOI:** 10.1080/07853890.2025.2573150

**Published:** 2025-10-23

**Authors:** Jingjing Wang, Yuyue Hou, Jie Tan, Yajing Zhang, Ruolin Wu, Yanmei Han, Xiaojing Ren, Xiaotian Xia, Zairong Gao

**Affiliations:** ^a^ Department of Nuclear Medicine, Union Hospital, Tongji Medical College, Huazhong University of Science and Technology, Wuhan, China; ^b^ Department of Medical Imaging, Henan Provincial People’s Hospital, Zhengzhou, China; ^c^ Hubei Province Key Laboratory of Molecular Imaging, Wuhan, China; ^d^ Key Laboratory of Biological Targeted Therapy, The Ministry of Education, Wuhan, China; ^e^ Department of Breast and Thyroid Surgery, Union Hospital, Tongji Medical College, Huazhong University of Science and Technology, Huazhong, China

**Keywords:** Clinical outcomes, differentiated thyroid cancer, radioactive iodine therapy, stimulated thyroglobulin

## Abstract

**Background:**

The incidence of differentiated thyroid cancer (DTC) has been increasing, highlighting the need for reliable predictors of treatment response. This study aimed to assess the prognostic value of stimulated thyroglobulin (sTg) levels before and after the first ^131^I treatment in DTC patients without distant metastases.

**Methods:**

Sixty patients were classified into excellent response (ER) and non-excellent response (NER) groups based on a comprehensive evaluation of imaging findings, sTg, suppressed Tg and other parameters. Clinical and pathological variables were analyzed using univariate and multivariate logistic regression. Receiver operating characteristic (ROC) curve analysis was used to determine optimal cut-off values for pre-^131^I sTg levels and for the rate of sTg decline after the first ^131^I therapy.

**Results:**

Both pre-^131^I sTg level (OR: 3.010, 95% CI: 1.004–9.029, *p* = 0.049) and the rate of sTg decline (OR: 0.756, 95% CI: 0.590–0.968, *p* = 0.026) were identified as independent predictors of clinical outcomes. The optimal threshold for pre-^131^I sTg was 14.55 μg/L (sensitivity: 95.0%, specificity: 67.5%, AUC: 0.804), and for the rate of sTg decline, 44.75% (sensitivity: 70.0%, specificity: 97.5%, AUC: 0.889). A prognostic nomogram was developed incorporating sex, age, pre- and post-^131^I sTg levels, T and N stages, tumor size, and thyroiditis.

**Conclusion:**

Lower pre-^131^I sTg levels and/or a greater rate of sTg decline after the first ^131^I treatment are associated with more favorable clinical outcomes. The proposed nomogram may assist clinicians in optimizing treatment decisions and stratifying follow-up strategies for patients with DTC.

## 1. Introduction

Thyroid cancer (TC) is the most common malignant tumour in the endocrine system. In recent years, with the widespread use of various imaging tests, fine-needle aspiration biopsy of thyroid nodules, and advancements in diagnostic techniques, the incidence rate of thyroid cancer have been increasing annually [1–3]. Differentiated thyroid cancer (DTC) is the most predominant type of thyroid cancer, accounting for over 90% of all cases [4–8]. Most DTC patients have low-grade malignancies, with slow disease progression and a favorable prognosis after comprehensive treatment. However, there remains a risk of local recurrence and distant metastasis, with nearly 10% to 30% of DTC patients experiencing recurrence or distant metastasis during long-term follow-up after initial treatment. Approximately 8% of those with local recurrence die from cancer, while the tumor-related mortality rate among patients with distant metastasis is around 50% [9].

The treatment efficacy assessment system, proposed by the 2015 American Thyroid Association (ATA) guidelines, has been widely recommended for evaluating the post-treatment outcomes of DTC patients, following surgery, thyroid-stimulating hormone suppression therapy, and initial ^131^I treatment [10]. This system considers multiple factors, including clinicopathological characteristics, postoperative serum markers such as thyroglobulin (Tg) and stimulated thyroglobulin (sTg), and various imaging modalities like whole-body scintigraphy, localized tomography, CT, MRI, PET/CT, and bone scans after ^131^I treatment. Treatment responses are classified as excellent response (ER), indeterminate response (IDR), biochemical incomplete response (BIR), and structural incomplete response (SIR).

Serum sTg refers to the Tg level measured after a period of thyroxine withdrawal or after recombinant thyroid stimulating hormone(TSH) stimulation. Numerous studies have demonstrated that the sTg level before the first postoperative ^131^I treatment is an effective predictor of prognosis in DTC patients [11–16]. Therefore, the pre-^131^I sTg level holds significant clinical value in assessing residual thyroid tissue, current disease status, and guiding treatment decisions, serving as a sensitive marker for detecting recurrence or metastasis in DTC patients [17]. However, current prognostic tools relying primarily on pre-^131^I sTg levels have limitations. Their predictive accuracy can be affected by factors such as residual thyroid tissue, serum TSH variability, and the presence of thyrotropin receptor antibodies (TRAb) [18–20]. Moreover, they often lack sufficient sensitivity and specificity for long-term outcome prediction, which complicates the early identification of patients at high risk for disease progression.

Given these challenges, there is a critical need to develop improved predictive methods that incorporate additional early clinical and pathological parameters to better assess disease regression and prognosis in DTC patients. In this study, we specifically included DTC patients without distant metastases to focus on a more homogeneous patient population. Patients without distant metastasis generally have a better baseline prognosis, and the evaluation of serum stimulated sTg dynamics after first ^131^I treatment can more accurately reflect residual disease activity and treatment response. In this study, DTC patients without distant metastases were retrospectively analyzed to explore the relationship between pre-^131^I sTg levels and the rate of sTg decline after the first ^131^I treatment. The rate of sTg decline after the first ^131^I treatment is an important parameter to study in conjunction with pre-^131^I sTg levels because it reflects the dynamic response of residual thyroid tissue or tumor cells to therapy. While the pre-^131^I sTg provides a snapshot of disease burden before treatment, the rate of decline captures the effectiveness of ^131^I ablation and may serve as an early indicator of tumor aggressiveness or treatment sensitivity. Patients with a slower or insufficient decline in sTg levels post-treatment are more likely to harbor persistent or recurrent disease, indicating a poorer prognosis. Thus, combining baseline sTg measurements with their post-treatment trajectory offers a more comprehensive assessment of disease status and improves prognostic accuracy. Our goal was to evaluate early clinical regression based on these parameters and to identify patients with poor prognoses, providing a basis for further clinical interventions. For patients with favorable prognoses, reduced follow-up frequency and intervention could be considered, potentially alleviating the economic and psychological burden on patients [21].

To address the complexity of prognostic factors and improve individualized risk prediction, we constructed a predictive nomogram integrating pre-^131^I sTg levels, the rate of sTg decline post-treatment, and other key clinicopathological features. Nomograms are widely recognized as practical and effective tools in clinical oncology for providing personalized risk assessments by combining multiple prognostic variables into a single, user-friendly graphical model [22]. Compared to traditional staging systems or single-factor predictors, nomograms generally offer superior predictive accuracy and better calibration, facilitating more precise clinical decision-making tailored to individual patient risk profiles.

## 2. Materials and methods

### 2.1. Study subjects and inclusion criteria

In this study, clinical data were retrospectively collected from 67 patients with DTC who underwent ^131^I treatment without distant metastasis at Department of Nuclear Medicine, Union Hospital affiliated to Tongji Medical College of Huazhong University of Science and Technology, between March 2019 and June 2022. Among them, 7 cases were excluded due to incomplete follow-up data, resulting in 60 patients with available data. The cohort included 26 males and 34 females, with ages ranging from 24 to 59 years (mean age: 38.78 ± 9.03 years), and a follow-up period ranging from 15 to 54 months. The collected data included gender, age at diagnosis, body mass index (BMI), pathologic subtype, maximum tumour diameter, tumour multifocality, extra-glandular invasion, extra-nodal lymph node invasion, T-stage and N-stage, total dose of ^131^I treatment, the pre-^131^I sTg level, the post-^131^I sTg level, value of sTg decrease after the first ^131^I treatment, sTg decrease rate, concomitant thyroiditis, and other clinicopathological characteristics.

Inclusion criteria were: (1) total bilateral thyroidectomy with pathologically confirmed DTC; (2) absence of distant metastasis; (3) sTg level ≥1.0 μg/L after initial treatment; (4) complete clinical data; and (5) a follow-up period of at least 1 year.

Exclusion criteria were: (1) imaging or pathology indicating distant metastasis; (2) positive TgAb; (3) incomplete clinical data; and (4) presence of other malignant tumour.

This retrospective study was approved by the Ethics Committee of Union Hospital, Tongji Medical College, Huazhong University of Science and Technology (NO. 2023569), and the requirement for obtaining written informed consent from the patients was waived.

### 2.2. Methods

Serological analysis of thyroid function was performed using a fully automated electrochemiluminescence immunoassay analyzer (Roche, Switzerland, cobas e801), with a Tg detection range of ≥0.04 μg/L. When Tg values were below 0.04 μg/L, 0.04 μg/L was used as the statistical value. The reagents used included streptavidin-coated magnetic microparticles, biotinylated anti-thyroglobulin antibody, and ruthenium-labeled anti-thyroglobulin antibody, all provided by Roche Diagnostics. Post-^131^I therapy whole-body imaging and regional tomography were conducted using a SPECT/CT instrument (670Plo) manufactured by GE Healthcare, USA. The Na^131^I stock solution was provided by Atomic High-Tech Co.

All patients underwent total thyroidectomy, and postoperatively patients were treated with RAI under thyroid hormone withdrawal (THW). THW is thyroid hormone withdrawal within 4 weeks prior to radioactive iodine (RAI) administration. Measurements of sTg and anti-Tg antibodies were taken within 1 week before RAI treatment. RAI whole-body scintigraphy was performed within 3–4 days after RAI to confirm the absence of structural disease or RAI uptake of thyroid residues; 6–12 months after the first RAI administration, sTg and neck ultrasound were performed. Afterwards, patients were followed up by clinical examination, laboratory tests, and relevant imaging.

### 2.3. Definition of clinical indicators

Tumour multifocality: according to the number of cancer foci in the pathology report of DTC patients, they are classified as unifocal DTC or multifocal DTC. Unifocal refers to only 1 cancer foci; multifocal refers to the number of cancer foci ≥ 2.

Maximum tumour diameter: defined as the diameter of the largest cancerous lesion in the pathology report.

TgAb positive: defined as TgAb > 115 IU/ml.

The postoperative pathological findings were used to determine whether the patient had intraglandular spread of thyroid gland, extra-thyroid gland invasion, lymph node metastasis, extra-lymph node invasion, and tumour staging. Tumour staging was referred to the 8th edition of the Alternate Joint Communications Center (AJCC) tumour staging system.

Initial treatment: refers to total thyroidectomy with or without cervical lymph node dissection, first ^131^I treatment, and TSH suppression therapy.

The value of sTg decrease after the first ^131^I treatment was calculated as the difference between the sTg level before and after the first ^131^I treatment.

The rate of sTg decreases after the first ^131^I treatment was calculated as:

$$\frac{\text{sTg before the first}{}^{131}I\text{treatment}-\text{sTg after the first}{}^{131}I\text{treatment}}{\text{sTg before the first}{}^{131}I\text{treatment }}\times 100\%$$

### 2.4. Efficacy assessment and grouping criteria

Referring to the 2015 ATA guidelines, patients were assessed for efficacy based on the results of the most recent review. The efficacy assessment could be divided into four categories, ER, IDR, BIR, and SIR, with the following criteria.

ER met all of the following criteria: (1) sTg < 1 μg/L or inhibitory Tg < 0.2 μg/L and negative TgAb;(2) Negative imaging.

IDR met all of the following criteria: (1) 1 μg/L ≤ sTg < 10 μg/L or 0.2 μg/L ≤ inhibitory Tg < 1 μg/L and stable or decreasing TgAb; (2) No structural lesions are found on neck ultrasound, lung CT, whole-body bone imaging, and other imaging tests, and whole-body imaging after ^131^I treatment suggests faint visualization of the original thyroid bed area.

BIR met all of the following criteria: (1) sTg ≥ 10 μg/L or inhibitory Tg ≥ 1 μg/L or an increasing trend in TgAb; (2) Negative imaging.

SIR: Imaging confirms the presence of structural or functional disease, and serum Tg/sTg or TgAb can be at any level.

Exclusion criteria for distant metastasis:

1. The presence of distant metastases confirmed pathologically by puncture or after surgery;
2. Exclusion of localized uptake or diffuse uptake in ^131^I whole-body and/or localized tomographic images due to physiological iodine uptake or contamination, with or without other positive imaging tumour indications, with or without elevated serum Tg/sTg levels;
3. Negative ^131^I whole-body imaging and localized tomography, but other imaging suggestive of positive tumour indications with elevated serum Tg/sTg levels.

The serological and imaging findings of the last follow-up of the included patients were evaluated for clinical regression and divided into the ER group (*n* = 20) and the NER group (*n* = 40), in which IDR, BIR, and SIR were uniformly attributed to the NER group.

### 2.5. Statistical analysis

Using SPSS 27.0 statistical software, the general clinicopathological characteristics of the first included patients were statistically analyzed, and the data information was tested for normal distribution, with measures conforming to the normal distribution expressed as mean ± standard deviation (±s), non-normally distributed measures expressed as median (inter-quartile range) (M (P25, P75)), and qualitative information expressed as frequency (percentage) (*n* (%)) Expressed. Univariate analyses were performed using the chi-square test, independent samples *t* test, and Mann–Whitney *U* rank-sum test as needed, and statistically significant factors were included in binary logistic regression analyses to determine independent risk factors affecting the response to treatment; receiver operating characteristic (ROC) curves were used to determine the sTg level before the first ^131^I treatment and the rate of decline of sTg after the first ^131^I treatment. ROC curves were used to determine sTg levels before the first ^131^I treatment and the rate of decrease in sTg after the first ^131^I treatment to predict the value of response to ^131^I treatment; *p* < 0.05 indicates statistically significant differences. Using R language version 4.4.1, the rms package was employed to construct a nomogram for predicting the likelihood of clinical outcomes based on clinical indicators.

## 3. Results

### 3.1. Univariate analysis of clinicopathological characteristics and clinical outcomes

The 60 patients could be divided into ER and NER groups based on the efficacy assessment of the follow-up results, including 20 patients in the ER group and 40 patients in the NER group. The results of the univariate analysis showed that the patients in the 2 groups showed a significant increase in the total dose of ^131^I treatment (*U* = 164.000, *p* < 0.001), the pre-^131^I sTg level (*U* = 157.000, *p* < 0.001), the post-^131^I sTg level (*U* = 44.000, *p* < 0.001), and the rate of sTg decline after the first ^131^I treatment (*U* = 89.000, *p* < 0.001) showed statistically significant differences in gender (χ^2^ = 0.543, *p* = 0.461), age at diagnosis (*t* = –1.428, *p* = 0.159), BMI (*U* = 377.000, *p* = 0.718), pathologic subtype (*t* = 0.501, *p* = 0.618), maximal diameter of the tumour (*U* = 647.500, *p* = 0.98), and tumour multifocality (χ^2^ = 0.049, *p* = 0.825), extra-glandular invasion of the tumour (χ^2^ = 1.026, *p* = 0.311), extra-nodal invasion of the lymph nodes (χ^2^ = 0.240, *p* = 0.624), T stage (χ^2^ = 4.058, *p* = 0.254), N stage (χ^2^ = 2.045, *p* = 0.360), the value of the decrease in sTg after the first ^131^I treatment (*U* = 380.000, *p* = 0.754), and concomitant thyroiditis (*t* = –1.252, *p* = 0.216), the difference was not statistically significant (Table 1).

**Table 1.** One-way analysis of general clinicopathological characteristics of patients in ER and NER groups.

General characteristics		ER group	NER group	χ^2^*/U*/*t*	*P*
Sex *n* (%)	Male	10 (50.0)	16 (40.0)	0.543	0.461
	Female	10 (50.0)	24 (60.0)		
Age at diagnosis (±s)/years		36.45 ± 9.83	39.95 ± 8.49	−1.428	0.159
BMI/kg/m^2^		25.28 (20.37, 26.72)*	24.48 (22.01, 26.61)*	377.000	0.718
Pathologic subtype (%)	Classic	19 (95.0)	39 (97.5)	0.501	0.618
	Follicular subtype	1 (5.0)	1 (2.5)		
Maximum tumour diameter/cm		1.67 (0.90, 2.00)*	1.57 (0.80, 2.15)*	647.500	0.980
Tumour multifocality *n* (%)	Unifocal	4 (20.0)	9 (22.5)	0.049	0.825
	Multifocal	16 (80.0)	31 (77.5)		
Extra-glandular invasion *n* (%)	Yes	4 (20.0)	13 (32.5)	1.026	0.311
	No	16 (80.0)	27 (67.5)		
Extra-nodal invasion *n* (%)	Yes	4 (20.0)	6 (15.0)	0.240	0.624
	No	16 (80.0)	34 (85.0)		
T staging *n* (%)	T1	13 (65.0)	21 (52.5)	4.058	0.254
	T2	2 (10.0)	6 (15.0)		
	T3	4 (20.0)	4 (10.0)		
	T4	1 (5.0)	9 (22.5)		
N staging *n* (%)	N0	1 (5.0)	0 (0.0)	2.045	0.360
	N1a	5 (25.0)	10 (25.0)		
	N1b	14 (70.0)	30 (75.0)		
the pre-^131^I sTg level/μg/L		7.97 (3.71, 11.73)*	41.43 (10.38, 4.43)*	157.000	<0.001
the post-^131^I sTg level/μg/L		3.33 (1.39, 4.29)*	32.29 (11.53, 40.85)*	44.000	<0.001
Total ^131^I treatment dose/mCi		274.50(250, 300)*	329.75 (300, 350)*	164.000	<0.001
Decrease in sTg after first ^131^I treatment/μg/L		3.26 (1.84, 5.39)*	3.28 (−0.49, 10.45)*	380.000	0.754
the rate of sTg decline after the first ^131^I treatment		53.46% (35.83%, 76.28%)*	−15.90% (−2.55%, 30.00%)*	89.000	<0.001
concomitant thyroiditis (%)	Yes	0 (0.0)	3 (7.5)	−1.252	0.216
	No	20 (100.0)	37 (92.5)		

*Note: Data presentation: median (inter-quartile range) (M (P25, P75)).

*ER* excellent response, *NER* non-excellent response.

**Figure 1.**
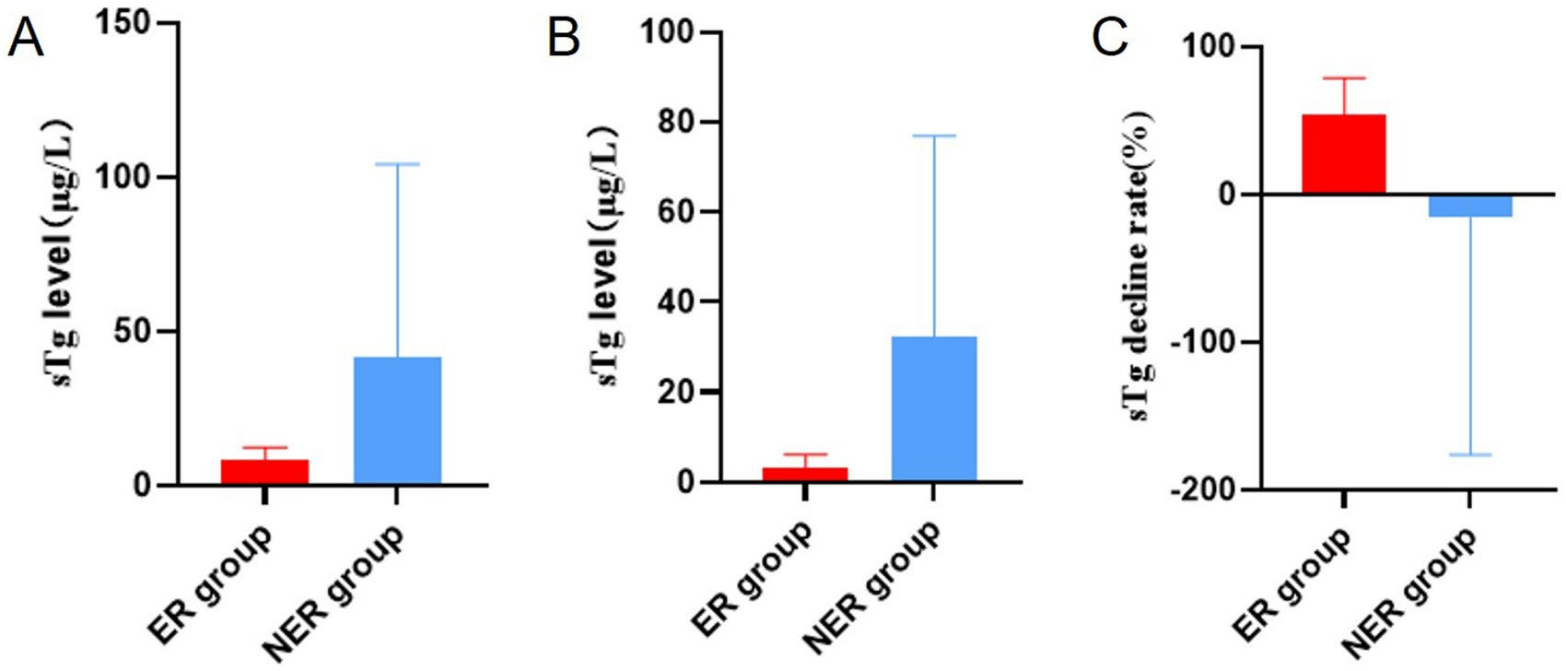
sTg levels before and after the first ^131^I treatment and the rate of sTg decrease after the first ^131^I treatment in the ER and NER groups. (A) sTg level before the first ^131^I treatment in ER group and NER group. (B) sTg level after the first ^131^I treatment in ER group and NER group. (C) Rate of sTg decrease after the first ^131^I treatment in ER group and NER group.

During the follow-up period, no patients experienced tumor-related specific mortality, nor did any patients experience adverse effects. 40 patients still did not meet the criteria for ER at the final follow-up, i.e. NER status. 20 patients had a good response to treatment at the final follow-up, i.e. ER status. The sTg levels before and after the first ^131^I treatment and the rate of sTg decrease after the first ^131^I treatment in the ER and NER groups are shown in Figure 1.

During the follow-up period, three DTC patients developed clinical recurrence after two ^131^I treatments, and the pathology after reoperation confirmed that local lymph node metastasis was present in all three cases. These three patients were classified as the NER group because the number of cases with local metastases was too small to assess the differences in the clinicopathological characteristics of the patients with metastases.

### 3.2. Multivariate analysis of clinicopathological characteristics and clinical outcomes

The results of binary logistic regression analysis showed that the sTg level before the first ^131^I treatment (OR: 3.01, 95% CI: 1.004–9.029, *p* = 0.049), the rate of decrease of sTg after the first ^131^I treatment (OR: 0.756, 95% CI: 0.590–0.968, *p* = 0.026) were correlated with the patients’ prognosis. The total dose of ^131^I treatment (OR: 1.036, 95% CI: 0.990–1.084, *p* = 0.123), and sTg level after the first ^131^I treatment (OR: 0.320, 95% CI: 0.101–1.011, *p* = 0.052) were not associated with patients’ prognosis (Table 2).

**Table 2.** Multifactorial analysis of general clinicopathological characteristics and clinical outcomes.

	*P* value	OR value	95% confidence interval	
			Lower limit	Upper limit
sTg level before first ^131^I treatment	0.049	3.010	1.004	9.029
sTg level after first ^131^I treatment	0.320	0.052	0.101	1.011
Total dose of ^131^I treatment	0.123	1.036	0.990	1.084
Rate of sTg decrease after first ^131^I treatment	0.026	0.756	0.590	0.968

### 3.3. ROC curve analysis

#### 3.3.1. Prediction of excellent response by sTg level before the first ^131^I treatment

**Figure 2.**
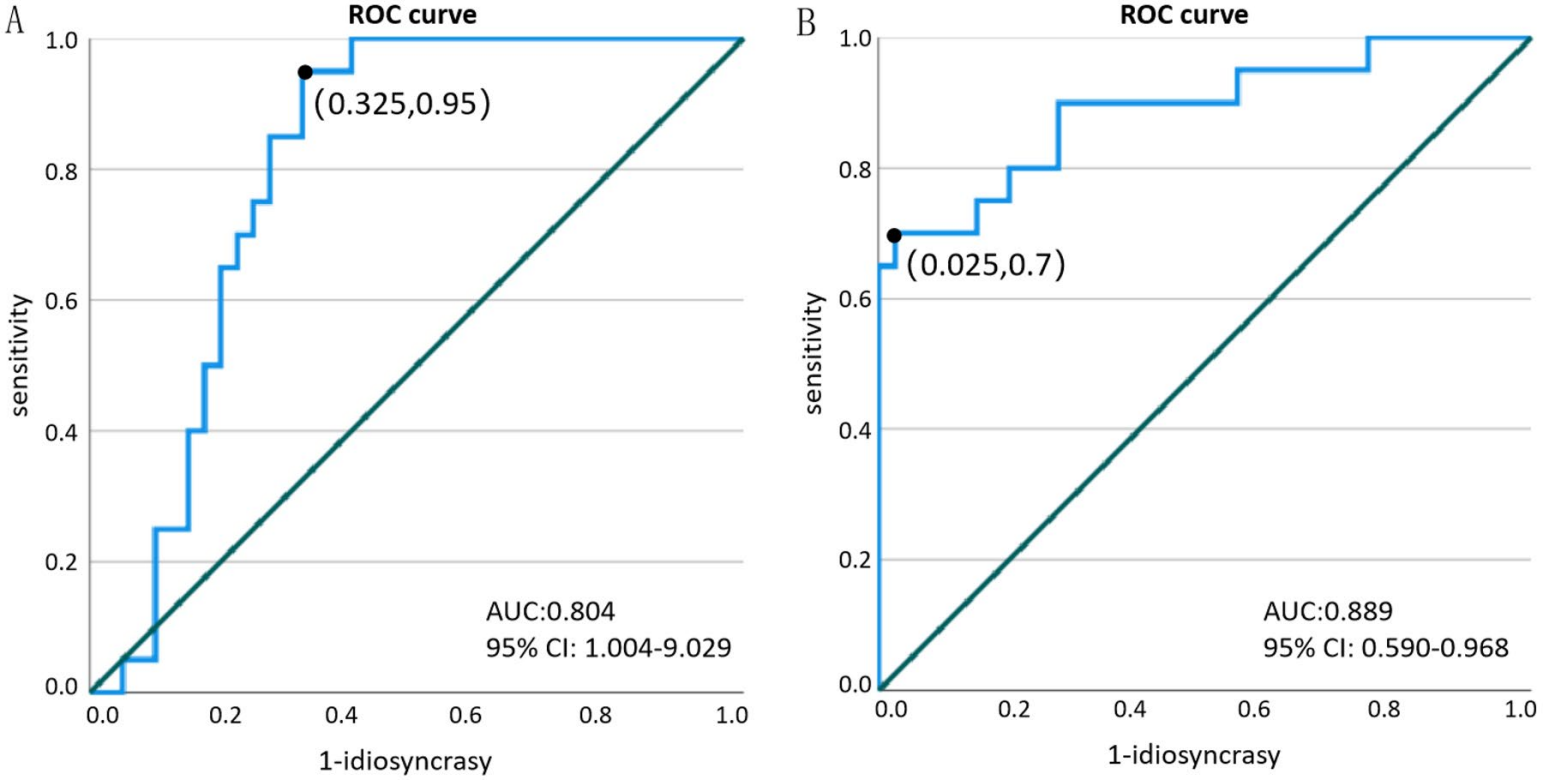
ROC curve of sTg level on ER before first ^131^I treatment and sTg decrease rate against ER after first ^131^I treatment. (A) ROC curve of sTg level on ER before first ^131^I treatment. (B) ROC curve of sTg decrease rate against ER after first ^131^I treatment.

**Figure 3.**
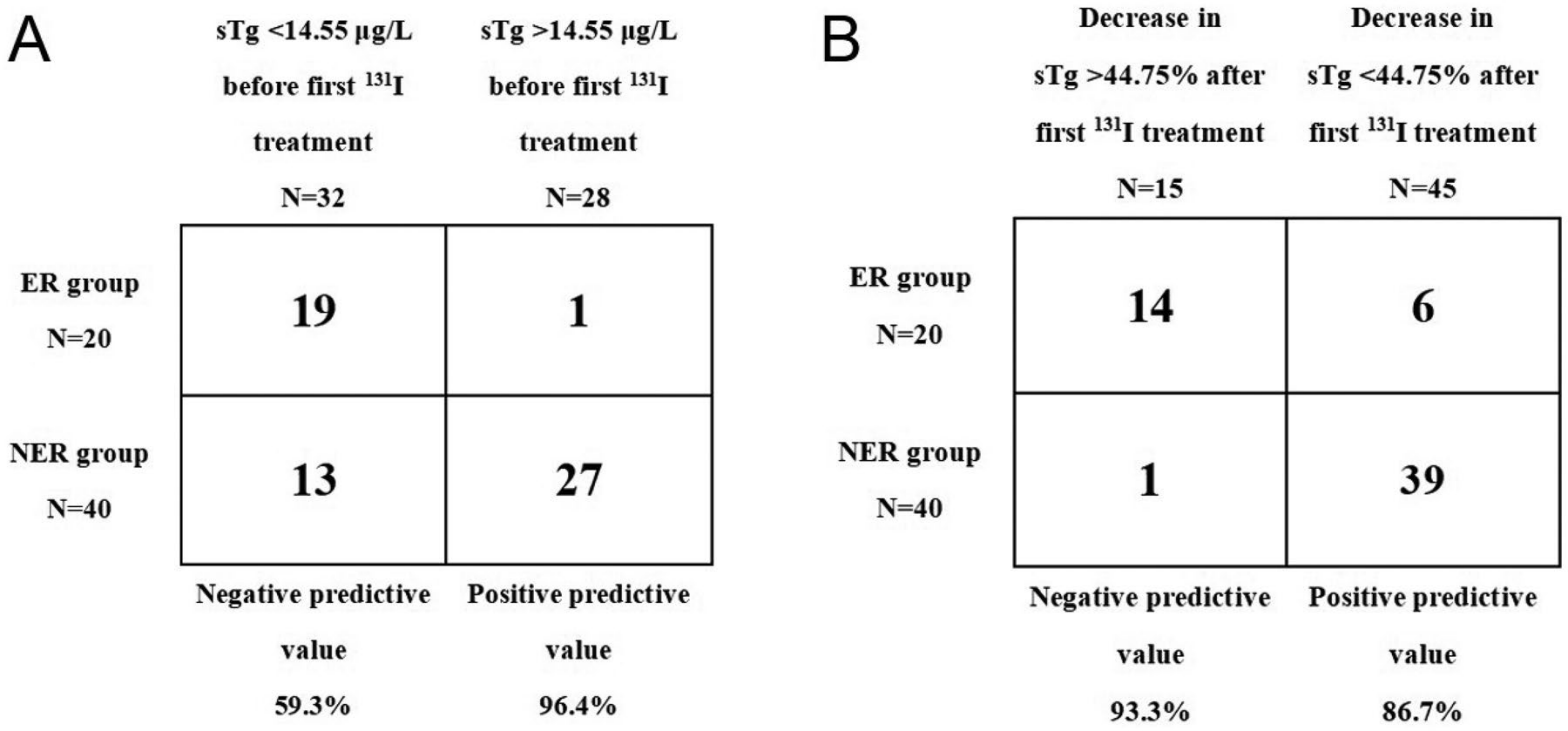
Positive and negative predictive values of sTg level before first ^131^I treatment and sTg decrease rate after the first ^131^I treatment. (A) Positive and negative predictive values of sTg level before first ^131^I treatment. (B) Positive and negative predictive values of sTg decrease rate after the first ^131^I treatment.

The median value of sTg level before the first ^131^I treatment was 7.97 (2.23, 16.00) μg/L in the ER group and 41.43 (1.87, 341.00) μg/L in the NER group, and the difference between them was statistically significant, and the sTg level before the first ^131^I treatment in the NER group was significantly higher than that in the ER group, indicating that The higher the sTg level before the first ^131^I treatment, the worse the prognosis. The ROC curves of sTg level before the first ^131^I treatment and ER group were established (Figure 2A), and the area under curve (AUC) was 0.804 (95% CI: 1.004–9.029). According to the results of the ROC curve, the Jordon index was greatest (0.625) when the sTg was 14.55 μg/L before the first ^131^I treatment, which corresponded to a sensitivity of 95.0% and a specificity of 67.5%, with a corresponding negative predictive value of 59.3% and a positive predictive value of 96.4% (Figure 3A).

#### 3.3.2. Predictive effect of sTg decrease rate on excellent response after the first ^131^I treatment

The median value of sTg decrease rate after the first ^131^I treatment was 53.46% (0%, 93.13%) in the ER group, and the median value of sTg decrease rate after the first ^131^I treatment was −15.9% (−980.21%, 45.81%) in the NER group, which was statistically significant, and the decrease in sTg decrease after the first ^131^I treatment in the ER group was significantly higher than that in the NER group, indicating that the more sTg decreased after the first ^131^I treatment, the better the prognosis. The ROC curves of the rate of sTg decrease after the first ^131^I treatment and ER were established (Figure 2B), and the AUC was 0.889 (95% CI: 0.590–0.968). According to the results obtained from the ROC curve, the Jordon index was greatest (0.675) when the change in sTg after the first ^131^I treatment was 44.75%, which corresponded to a sensitivity of 70.0%, a specificity of 97.5%, a negative predictive value of 93.3%, and a positive predictive value of 86.7% (Figure 3B).

### 3.4. Nomogram predictive model

**Figure 4.**
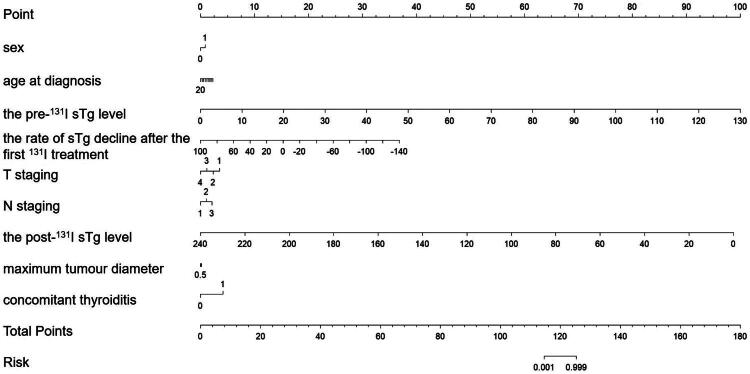
Nomogram predictive model.

Based on the above analysis, we selected nine factors, including sex, age at diagnosis, the pre-^131^I sTg level, the rate of sTg decline after the first ^131^I treatment, T staging, N staging, the post-^131^I sTg level, maximum tumour diameter, and concomitant thyroiditis, to make a nomogram for predicting clinical outcomes (Figure 4).

## 4. Discussion

Globally, the incidence of DTC is rising annually. Despite its generally favorable prognosis, some DTC patients experience recurrence or distant metastasis during follow-up. Early assessment for recurrence and identification of patients with poor prognoses are crucial for improving survival outcomes in DTC patients.

DTC originates from the follicular epithelial cells of the thyroid, retaining many of their functions during disease progression, such as expressing the sodium-iodide symporter (NIS) and secreting Tg. Thus, thyroid cancer cells continue to secrete Tg, similar to normal thyroid cells. After undergoing therapy, DTC patients are expected to have very low or undetectable levels of Tg/sTg. In this study, by analyzing the clinicopathological characteristics of DTC patients without distant metastasis, we confirmed that both pre-^131^I sTg levels and the rate of sTg decline after ^131^I treatment are valuable predictors of long-term clinical outcomes.

Some researchers have questioned the predictive value of pre-^131^I sTg levels due to factors like residual thyroid tissue, TSH, and TgAb levels [23]. However, several recent studies have shown that pre-^131^I sTg levels are reliable indicators of disease remission, persistence, or relapse in DTC patients. For example, a study of 166 children and adolescents with DTC showed that pre-^131^I sTg was an important prognostic factor for distant metastasis, with a sensitivity of 100% and specificity of 93% [24]. Similarly, Yixuan Li et al. found that pre-^131^I sTg was an independent prognostic factor for clinical regression in a study involving 135 intermediate-risk papillary thyroid cancer patients [25]. Picardo et al. reported consistent findings in a study of 243 high-risk DTC patients [26]. Additionally, a large meta-analysis of 50 studies involving around 4,000 patients demonstrated that pre-^131^I sTg has a negative predictive value of 94.2% for distant disease-free status [27]. Yang et al. [28] also found a correlation between higher pre-^131^I sTg levels and increased risk of recurrence.

In this study, we found that pre-^131^I sTg levels were significantly higher in the NER group compared to the ER group, with a statistically significant difference. This indicates that lower pre-^131^I sTg levels are associated with a higher likelihood of achieving ER status during long-term follow-up. The ROC analysis showed an AUC of 0.804, confirming that pre-^131^I sTg is an accurate predictor of ER status. These findings are consistent with previous studies [29].

Although pre-^131^I sTg levels have proven useful in predicting clinical outcomes, there is no universally agreed-upon threshold for predicting long-term regression. Various studies have proposed optimal thresholds ranging from 1 to 30 ng/mL [26,30–33]. In this study, ROC analysis revealed that a pre-^131^I sTg threshold of 14.55 μg/L had good predictive value for clinical regression, with a sensitivity of 95.0%, specificity of 67.5%, negative predictive value of 59.3%, and positive predictive value of 96.4%. This threshold provides a valuable reference for predicting clinical outcomes in patients with pre-^131^I sTg ≥ 1.0 μg/L and no distant metastasis after initial treatment. While pre-^131^I sTg levels demonstrated significant prognostic value in our study, it is worth noting that some statistical results, such as the association between pre-^131^I sTg and clinical outcome (*p* = 0.049), are close to the conventional threshold for significance. Given the multiple comparisons conducted, the potential for type I error cannot be excluded. Therefore, these findings should be interpreted with caution and validated in larger, independent cohorts to confirm their robustness.

Post-^131^I sTg levels are crucial for predicting recurrence in DTC patients. Kloos et al. identified a post-^131^I sTg threshold of <0.5 ng/mL, which predicted no recurrence within 3–5 years [34], while Castagna et al. suggested a threshold of <1.0 ng/mL [35]. However, for patients with pre-^131^I sTg ≥ 1.0 μg/L, post-^131^I sTg levels alone are insufficient to assess individual clinical regression.

In this study, we found that the rate of sTg decline after the first ^131^I treatment was significantly greater in the ER group than in the NER group, indicating that a larger decline in sTg is associated with better long-term clinical outcomes. The ROC analysis demonstrated an AUC of 0.889, suggesting high accuracy in predicting ER status. Furthermore, the rate of sTg decline was found to be an independent prognostic factor, underscoring its importance in individualized patient follow-up and treatment.

Although studies on the rate of sTg decline are limited, our ROC analysis showed that a decline rate of 44.75% had good predictive value, with a sensitivity of 70.0%, specificity of 97.5%, negative predictive value of 93.3%, and positive predictive value of 86.7%. A study by Bertrand Barres et al. suggested a decline rate of >60% as a predictor of clinical regression [36], which aligns with our findings. Nevertheless, more studies are needed to confirm the optimal threshold for sTg decline rates after ^131^I treatment.

The nomogram developed in this study serves as a valuable tool for clinicians to predict individualized outcomes in DTC patients by combining factors such as sex, age at diagnosis, pre-^131^I sTg level, rate of sTg decline after the first ^131^I treatment, T stage, N stage, post-^131^I sTg level, maximum tumor diameter, and concomitant thyroiditis. This visual and quantifiable method for assessing prognosis can help guide more personalized treatment strategies.

It is noteworthy that not all patients with elevated pre-^131^I sTg levels experience adverse clinical outcomes. Factors such as varying degrees of tumor differentiation, differences in iodine avidity, and individual immune responses may contribute to this inconsistency. Additionally, residual normal thyroid tissue or interference from TgAb may affect sTg measurements, potentially reducing the accuracy of prognostic assessment in certain cases. Recognizing these discrepancies underscores the necessity of incorporating multiple parameters, such as the sTg decline rate and clinicopathological features, into predictive models rather than relying solely on a single biomarker. The design of our nomogram, which integrates multiple factors, aims to capture this complexity, thereby enhancing the accuracy of individualized risk assessment.

This study innovatively examines the predictive impact of the decline rate of sTg following first ^131^I therapy on the clinical outcomes of patients with DTC who lack distant metastases. It calculates the optimal threshold value and demonstrates that the sTg decline rate after the first ^131^I treatment serves as a valid independent predictor for these patients. There is a lack of studies on the predictive value of the sTg decline rate after the first ^131^I treatment on the clinical outcome of patients with DTC without distant metastases, and this study fills the gap in this aspect to a certain extent. Meanwhile, from the perspective of DTC without distant metastasis, this study discussed the predictive effect of sTg level before the first ^131^I treatment on the clinical outcome of such patients, and calculated the optimal cut-off value, which provided a more meaningful reference value for the individualized clinical management decision of such patients. Practically, this enables tailored treatment protocols: patients identified as high-risk due to elevated pre-^131^I sTg and insufficient sTg decline can be prioritized for more aggressive monitoring, additional ^131^I therapy, or adjuvant treatments to prevent recurrence. Conversely, patients with low-risk profiles may benefit from de-escalated follow-up schedules and reduced intervention intensity, thereby minimizing unnecessary exposure to radiation, healthcare costs, and psychological stress. Furthermore, early identification of patients unlikely to achieve excellent response supports proactive clinical decision-making, allowing timely adjustments in therapy that may improve long-term outcomes. Ultimately, this approach promotes precision medicine by aligning treatment intensity with individual disease biology and response dynamics, improving survival while optimizing resource allocation.

There are some limitations to this study. First, the single-center retrospective design of this study carries potential selection bias, as the inclusion criteria and availability of complete clinical data may have favored patients with more regular follow-up or specific clinical characteristics, thereby limiting the generalizability of the findings. Second, many patients who underwent multiple ^131^I treatments had distant metastases or imaging suggestive of suspicious tumours, leading to a relatively small sample size. Third, due to the generally favorable prognosis and long survival of DTC patients, the follow-up period in this study was relatively short, and a longer follow-up is required for more comprehensive analysis. Fourth, due to time constraints, the nomogram model has not yet been put into the validation stage, and we will continue to improve it in the future.

To build upon our findings, future studies should focus on several key areas. First, prospective multicenter trials with larger and more diverse cohorts are needed to validate and generalize the predictive accuracy of the nomogram, especially across different ethnicities and treatment settings. Second, longer follow-up durations are essential to assess the nomogram’s utility in predicting not only early treatment response but also long-term recurrence and overall survival. Lastly, integration of emerging molecular and genetic markers with biochemical and clinical parameters may enhance personalized prognostication, warranting exploration in future predictive models.

## 5. Conclusion

Both the pre-^131^I sTg level and the rate of sTg decline after the first ^131^I treatment are associated with the clinical regression of DTC patients. Lower pre-^131^I sTg levels and greater rates of decline are linked to better prognoses, with optimal predictive values of 14.55 μg/L and 44.75%, respectively. The addition of a nomogram integrating the pre-^131^I sTg level and the rate of sTg decline after the first ^131^I treatment with other clinicopathological factors improves the prediction of clinical outcomes in DTC patients without distant metastasis. For high-risk patients, more proactive monitoring, additional ^131^I therapy, or adjuvant treatment can be prioritized to prevent recurrence. Conversely, low-risk patients may benefit from de-escalated follow-up protocols and reduced intervention intensity. Monitoring these indicators enables clinicians to adjust treatment plans dynamically, providing a foundation for more individualized patient care. The study demonstrates innovation, proving that further in-depth research is feasible and meaningful in the future.

## Data Availability

The datasets generated or analyzed during the study are available from the corresponding author on reasonable request.

## Abbreviations

DTC: Differentiated thyroid cancer
sTg: Stimulated thyroglobulin
ER: Excellent response
NER: Non-excellent response
ATA: American thyroid association
ROC: Receiver operating characteristic
TC: Thyroid cancer
Tg: Thyroglobulin
IDR: Indeterminate response
BIR: Biochemical incomplete response
SIR: Structural incomplete response
TRAb: Thyrotropin receptor antibodies
BMI: Body mass index
THW: Thyroid hormone withdrawal
RAI: Radioactive iodine
AUC: Area under curve
NIS: Sodium-iodide symporter
